# A novel mechanism of LIN-28 regulation of *let-7* microRNA expression revealed by in vivo HITS-CLIP in *C. elegans*

**DOI:** 10.1261/rna.045542.114

**Published:** 2015-05

**Authors:** Giovanni Stefani, Xiaowei Chen, Hongyu Zhao, Frank J. Slack

**Affiliations:** 1 Department of Molecular, Cellular and Developmental Biology, Yale University, New Haven, Connecticut 06405, USA; 2 Centre for Integrative Biology (CIBIO), University of Trento, 38123 Povo (TN), Italy; 3 Program in Computational Biology and Bioinformatics, Yale University, New Haven, Connecticut 06511, USA; 4 Department of Biostatistics, Yale School of Public Health, New Haven, Connecticut 06511, USA; 5 Department of Genetics, Yale School of Medicine, New Haven, Connecticut 06510, USA

**Keywords:** HITS-CLIP, LIN-28, RNA-binding, *let-7*, miRNA

## Abstract

The evolutionarily conserved gene *lin-28* encodes an RNA-binding protein and is an important regulator of the proper temporal succession of several developmental events in both invertebrates and vertebrates. At the cellular level, LIN-28 promotes stemness and proliferation, and inhibits differentiation, a feature best illustrated by its ability to induce pluripotency when ectopically expressed in human fibroblasts in combination with *NANOG, OCT4,* and *SOX2*. Mammalian LIN28 functions in part by regulating processing of the *let-7* microRNA through a GGAG binding site in the pre-*let-7*’s distal loop region. However, many human and animal *let-7* precursors lack the GGAG binding motif. In order to dissect the molecular mechanisms underlying its biological functions in a living animal, we identified a map of LIN-28 interactions with the transcriptome by in vivo HITS-CLIP in *Caenorhabditis elegans*. LIN-28 binds a large pool of messenger RNAs, and a substantial fraction of the bona fide LIN-28 targets are involved in aspects of animal development. Furthermore, our data show that LIN-28 regulates the expression of the *let-7* microRNA by binding its primary transcript in a previously unknown region, revealing a novel regulatory mechanism.

## INTRODUCTION

The timing of development is tightly controlled in animals and involves a cascade of gene expression regulatory events (Moss 2007). Forward genetics in *Caenorhabditis elegans* has greatly contributed to the molecular identity of regulators involved in this timing pathway collectively referred to as heterochronic genes, encompassing, among others, transcription factors, RNA-binding proteins, and microRNAs (miRNA) (Ambros and Horvitz 1984; Ambros 1989; Moss 2007). *lin-28* is a heterochronic gene whose loss of function leads to precocious terminal differentiation and reduced number of seam cells, a specialized subpopulation of multipotent hypodermal skin cells (Ambros 1989). In addition, *lin-28* mutant animals display several other defects of early development: disrupted differentiation timing of neurons, and a nonfunctional, protruding vulva, which leads to an inability to lay eggs (Ambros 1989; Euling and Ambros 1996; Olsson-Carter and Slack 2010). The expression of *lin-28* is limited to the early phase of development, rapidly decreasing after the second larval molt, partially as a result of the regulatory action of the miRNA *lin-4* (Moss et al. 1997). *lin-28* interacts genetically with other heterochronic genes: The persistent expression of *lin-14* requires LIN-28, while the *lin-28* mutant phenotype can be suppressed by mutations in *lin-46* (Arasu et al. 1991; Pepper et al. 2004). Furthermore, mutation of *let-7* partially rescues the precocious differentiation of seam cells in *lin-28* mutants, and *lin-28* is required for the correct temporal expression of *let-7* (Reinhart et al. 2000; Johnson et al. 2003; Van Wynsberghe et al. 2011).

At the cellular and organismal level, the functions and pattern of expression of *lin-28* are, in broad terms, strikingly consistent between *C. elegans* and vertebrates. Like in nematodes, the mammalian orthologs of *lin-28* (*Lin28A* and *Lin28B*) are expressed during early developmental stages, mostly in cell populations undergoing active proliferation, and polymorphism of *LIN28B* are associated with variations in the timing of human development (Yang and Moss 2003; Yokoyama et al. 2008; Ong et al. 2009). Deletion of *Lin28A* causes reduced body size in mice, while its over-expression produces abnormally large animals (Zhu et al. 2011). Sequence polymorphism of *LIN28B* also affects body size in humans (Lettre et al. 2008). Furthermore, Lin28 affects glucose metabolism as documented in genetically modified mice (Zhu et al. 2011). The proliferative and antidifferentiation functions of *LIN28* are co-opted in a number of human cancers, where its expression is reactivated, resulting in more aggressive and rapidly growing tumors (Viswanathan et al. 2009). Similarly, these functional proprieties have been exploited for the induction of pluripotency in human fibroblasts, by the simultaneous transduction of LIN28, OCT4, SOX2, and NANOG (Yu et al. 2007).

These biological functions are likely to be largely achieved through the regulation of the expression of other genes, as LIN28's best discernible functional domains are a cold shock domain (CSD) and two CCHC-type zinc-finger (ZnF) domains, both well-known nucleic acid recognition motifs. The most fully characterized molecular function of Lin28 in vertebrates is the inhibition of the maturation of the miRNA *let-7* (Heo et al. 2008; Newman et al. 2008; Rybak et al. 2008; Viswanathan et al. 2008). There are two proposed mechanisms for LIN28-mediated regulation of *let-7* maturation in vertebrates. LIN28 inhibits the cytoplasmic, Dicer-mediated maturation step from pre-*let-7* to mature *let-7* and promotes its degradation via the addition of a short stretch of Uridine residues by Terminal Uridine transferase (Heo et al. 2009). Additionally, the relatively nucleus-enriched LIN28B inhibits the Drosha-mediated step of maturation from pri-*let-7* to pre-*let-7* (Newman et al. 2008; Viswanathan et al. 2008; Piskounova et al. 2011). Regardless of the regulatory mechanism, LIN28 binds sequences in the terminal loop of pri- or pre-*let-7* in mammals (Newman et al. 2008; Piskounova et al. 2008; Heo et al. 2009). X-ray crystallography and NMR studies show that the Zinc-finger domains of LIN28 recognize a “GGAG” motif in a sequence-specific manner, through hydrogen bonds between the amino acid residues and the edge of the bases (Nam et al. 2011; Loughlin et al. 2012). Interestingly, not all copies of the 15 human *let-7* genes contain the GGAG motif in their loop and this motif is also absent in most invertebrate *let-7*s.

While forward *C. elegans* genetics has positioned *lin-28* in the heterochronic pathway and studies in cells in culture have revealed interactions with a number of mRNAs (Cho et al. 2012; Wilbert et al. 2012; Hafner et al. 2013), the molecular characterization of LIN-28 function in the context of the development of an entire organism is lacking. In order to obtain an exhaustive map of LIN-28 interactions with the transcriptome of developing *C. elegans*, we performed a copurification of RNA crosslinked in vivo to LIN-28, followed by its characterization by high-throughput sequencing, a technique known as HITS-CLIP (Licatalosi et al. 2008). Our results show that LIN-28 interacts with a large number of mRNAs involved in animal development, including two that were known to interact functionally with *lin-28* from genetic studies. Additionally, our study reveals that LIN-28 regulates *let-7* maturation by interacting with a novel site in pri-*let-7*, distinct from the terminal loop characterized in mammals.

## RESULTS

### Mapping of HITS-CLIP libraries and binding sites identification

**FIGURE 1.**
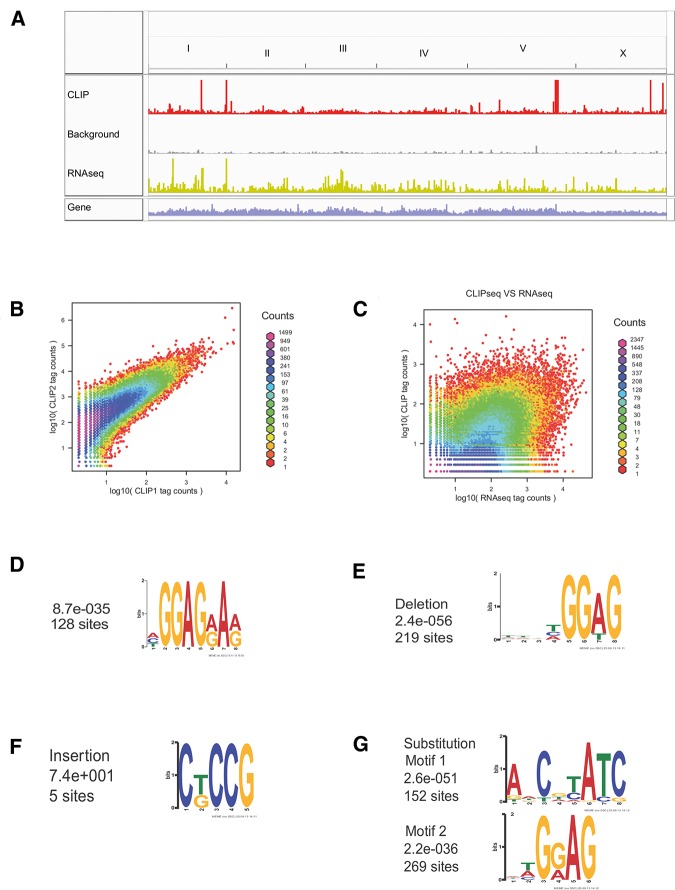
(*A*) A genome-wide view of LIN-28 interactions with the *C. elegans* transcriptome. Reads from a representative CLIP experiment, a matching background control, and an Input (RNA-seq) control are displayed in Integrated Genome Viewer (IGV) (Robinson et al. 2011). Number of reads in each line was normalized by total number of mapped reads. (*B*) Reproducibility of two CLIP experimental trials. (*C*) Correlation between read depth in CLIP samples and RNA abundance (RNA-seq). (*D*) Motif discovered by the Multiple EM for Motif Elicitation (MEME) tool within the binding sites data set defined by peak analysis. (*E*) Motif discovered by MEME analysis within the binding sites data set defined by deletions. (*F*) Motif discovered by MEME analysis within the binding sites data set defined by insertions. (*G*) Motifs discovered by MEME analysis within the binding sites data set defined by substitutions.

LIN-28 is highly expressed in the *C. elegans* L1 stage and functions at the L1 molt to prevent precocious expression of L3 fates in seam cells. Living late L1 stage animals were exposed to UV light to cross-link proteins and RNAs in situ (see Materials and Methods). In vivo crosslinked RNA was copurified with a rescuing LIN-28 fused to HA tag and characterized by high-throughput sequencing. As a control for background, we isolated and prepared samples in an identical manner from a strain lacking the HA tag. Supplemental Table S1 shows that we obtained 6,727,518 reads from CLIPseq 1 and 206,665,887 reads from a second biological replicate, CLIPseq 2. The reads from the CLIP experiments were mapped to the *C. elegans* genome version WS190/ce6 by Novoalign (http://www.novocraft.com). About 75% of reads generated by HITS-CLIP (5,087,544 for CLIPseq 1 and 156,886,622 for CLIPseq2) could be mapped to the *C. elegans* genome yielding a complete snapshot of LIN-28/transcriptome interactions at the L1 stage (Fig. 1A). The read depth distribution by 150-bp windows of exon regions between experimental trials shows a high level of reproducibility with a correlation coefficient of 0.803 (Fig. 1B). The relatively poor correlation (0.455) between read depth in CLIP samples and RNA abundance (RNA-seq) reveals that CLIP captures specific protein–RNA interactions and is not overly affected by transcript abundance; however, a correlation level of 0.455 also indicates that RNA-seq can be treated as a good matching control for exon regions (Fig. 1C).

We identified LIN-28 binding sites by a novel CLIP data analysis pipeline that relies on both peak analysis and crosslinking induced mutation site (CIMS) analysis (Kishore et al. 2011; Zhang and Darnell 2011). For peak analysis, we devised a parametric model based on combination of dynamic Poisson and negative binomial regression models to identify and quantify binding events (see Materials and Methods). The CIMS analysis is made possible by the occurrence of mutations in the reverse transcription of RNA molecules that had been crosslinked to protein, likely due to residual peptides disrupting the fidelity of cDNA synthesis by Reverse Transcriptase (Zhang and Darnell 2011). This analysis revealed that LIN28 binds an excess of two thousands mRNA sites in vivo (Supplemental Tables S2, S3). Within this data set of candidate target sequences, we searched for the presence of shared enriched motifs using the Multiple Expectation maximization for Motif Elicitation (MEME) algorithm (Bailey et al. 2009). In order to evaluate the consistency of motif identification between two analyses, we undertook MEME searches within the target sets obtained by peak analysis and CIMS separately. Within the peak analysis data set, we identified a top-scoring motif with length 8 bp with score 8.7 × 10^−035^ containing the GGAG quadruplet, similarly to the data sets generated in vertebrate cells (Fig. 1D; Cho et al. 2012). Within the target set obtained by CIMS, we evaluated separately the sets obtained by three types of mutations: deletions, insertions, and substitutions. The sequence tags identified by deletions presented motifs similar to the ones predicted based on peak analysis, a 6 bp motif containing GGAG (Fig. 1E). However, this pattern was not present in the sets generated on the basis of insertions (Fig. 1F). Within the binding sites identified by substitutions, a GGAG-containing element was identified alongside a different motif (Fig. 1G; Supplemental Fig. S1A). High motif enrichment in high confident deletions (∼900) and substitutions (approximately top 2000) of CLIP1 also shows that these two types of mutations contain relatively high proportion of CIMS; however, lower ranked substitutions might be diluted by sequencing errors and SNPs in the sample (Supplemental Fig. S1B). Thus, deletion (BH ≤ 0.05 listed in Supplemental Table S4) appears to be the primary mutation type induced by cross-linking to proteins in the CLIP protocol, but substitution (BH ≤ 0.05 listed in Supplemental Table S5) also contains a proportion of crosslinking information. Furthermore, CIMS analysis contributes significantly to pinpoint accurate sites of protein–RNA interactions, as the average length of binding site sequences from peak analysis is ∼300 nt (Supplemental Fig. S1C), while it is ∼40 nt for CIMS (Supplemental Fig. S1D).

**FIGURE 2.**
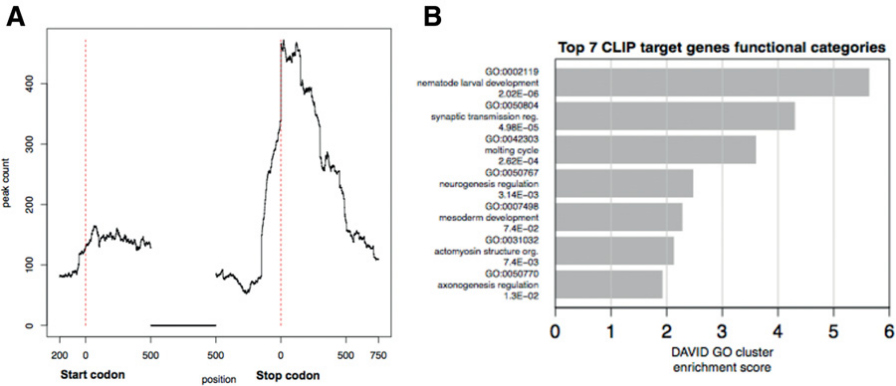
(*A*) LIN-28 binding site distribution within mRNA regions. The *x*-axis is the position between 200 bp upstream of start codons and 750 bp downstream from stop codons. The highest enrichment of LIN-28 binding sites is observed within 3′ UTRs. (*B*) Gene Ontology enrichment analysis for LIN-28 bona fide targets; top seven scoring clusters are shown. Clusters were defined using DAVID Gene Functional Annotation Clustering. GO BP (biological processes) “FAT” annotations and “highest” stringency were used. Clusters are annotated with representative GO terms and corresponding Benjamini–Hochberg FDR corrected *p*-values, and ranked by enrichment score.

The binding sites distribution within transcripts shows a marked under-representation in the 5′ UTR (3.96%) compared with coding sequence (56.52%) and 3′ UTR (39.52%) (Fig. 2A). Nonetheless, given that 3′ UTRs are on average shorter than coding sequences, the highest enrichment of CLIP tags per sequence length is observed in the former. For each region type (5′ UTR, CDS and 3′ UTR), we calculated an enrichment score based on

$${\text{EnrichScore}}_{\text{region}}=\frac{\text{no. of peaks in region}}{\text{length of region}/\text{no. of genes}},\;\text{region}:\;5\prime \;\text{UTR},\;3\prime \;\text{UTR or CDS}.$$

The score for each region type is 5′ UTR 0.700, CDS 1.495 and 3′ UTR 1.864. Thus peaks are mostly enriched at 3′ UTRs. Notably, the highest abundance of peaks within coding regions is also near their 3′ ends (Fig. 2A).

Overall, the sole enrichment within our data set of the GGAG motif, which has been extensively validated through mutational and structural studies in the context of Lin28 binding to *let-7* terminal loop, is a strong indication of the validity of the bona fide target sequences identified by CLIP.

### HITS-CLIP identifies a large set of LIN-28 target transcripts

The analysis of the CLIP data set identifies an excess of 2000 in vivo LIN-28 binding sites (Supplemental Tables S2, S3). A search for over-represented terms in the Gene Ontology (GO) database shows a notable enrichment of biological process terms related to animal development (Fig. 2B; Supplemental Table S6). Nematode larval development is the most highly enriched category, consistent with the well-established role of *lin-28* as a regulator of postembryonic animal development.

**FIGURE 3.**
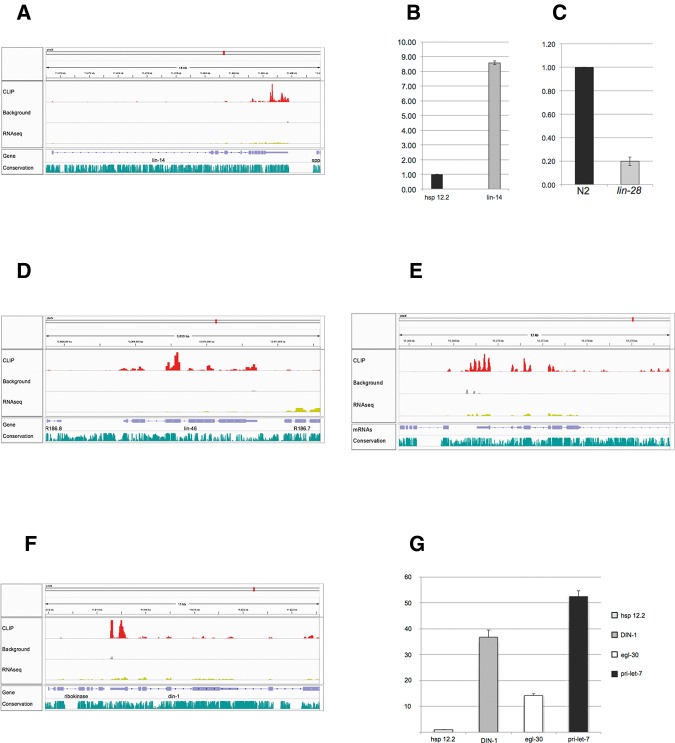
LIN-28 interacts with heterochronic genes mRNAs. (*A*) Map of LIN-28 interactions with the *lin-14* mRNA visualized by IGV. The number of reads in each track was normalized by the total number of mapped reads. (*B*) RNA-coimmunoprecipitated with LIN-28 was analyzed by RT-qPCR with primers for *hsp-12.2* (negative control) and *lin-14*. The abundance of these mRNAs in the RIP sample was normalized to their abundance in the input material. (*C*) The abundance of *lin-14* in wild-type animals (N2) and *lin-28* mutants, detected by qPCR. (*D*) Map of LIN-28 interactions with *lin-46* mRNA. (*E*) Map of LIN-28 interactions with *kin-20* mRNA. (*F*) Map of LIN-28 interactions with *din-1* mRNA. (*G*) RIP analysis of interactions between LIN-28 and *hsp-12.2* (negative control), *din-1*, *egl-30* mRNAs and *let-7* primary transcript (pri-*let-7*).

The heterochronic pathway has been extensively characterized in *C. elegans* by epistasis experiments over more than two decades. Within this pathway, *lin-28* is known to interact genetically with *lin-14* (ranked 220 in our list), a determinant of early phases of development. Immunofluorescence experiments have shown that *lin-28* positively regulates *lin-14* protein levels (Arasu et al. 1991). Our data show that LIN-28 interacts with *lin-14* mRNA, mostly within the 3′ UTR (Fig. 3A). This interaction was confirmed in independent experiments by RNA-coimmunoprecipitation (RIP) followed by qPCR (Fig. 3B). Furthermore, the abundance of *lin-14* mRNA is decreased in *lin-28* mutants, suggesting that the previously documented positive effect of *lin-28* on *lin-14* protein levels is the result of an overall stabilizing effect on *lin-14* mRNA (Fig. 3C).

Forward genetic screens have identified *lin-46* (ranked 604 in our list), another heterochronic gene, as a suppressor of *lin-28* (Pepper et al. 2004). Our CLIP experiment documents extensive interactions of LIN-28 with *lin-46* mRNA, both within the coding sequence and the 3′ UTR, suggesting that at least part of the functional interaction is caused by a physical interaction between LIN-28 protein and mRNA (Fig. 3D). LIN-28 also binds the mRNA of the developmental timing kinase gene *kin-20*, homolog of *Drosophila* clock gene “doubletime” (position 1013) (Fig. 3E; Supplemental Table S3; Banerjee et al. 2005). In addition, LIN-28 interacts with its own mRNA (ranked 1024 in our list, Supplemental Table S3) suggesting that LIN-28 autoregulates its own expression.

Furthermore, HITS-CLIP identified an interaction of LIN-28 with the mRNA of *din-1* (ranked 20 in our list), a heterochronic gene implicated, like *lin-28*, in the regulation of *let-7* expression (Ludewig et al. 2004; Bethke et al. 2009). Unlike LIN-28, which inhibits *let-7* maturation post-transcriptionally, DIN-1 forms a transcriptionally silent complex with the nuclear receptor DAF-12, inhibiting the transcription of pri-miRNAs of the *let-7* family (Bethke et al. 2009). LIN-28 binds with the 3′ UTR of *din-1* mRNA, an interaction that was confirmed in separate RIP-qPCR experiments (Fig. 3F,G).

These data show that LIN-28 interacts with a large population of transcripts during *C. elegans* development. While the functional implications of the vast majority of these interactions remain currently not understood and will be the subject of future investigation, a subset of the identified targets are known regulators of the timing of animal development, which, in the case of *lin-14* and *lin-46*, were known to interact genetically with *lin-28*.

In addition, a subset of the LIN-28 interacting genes are shared with those interacting with the homologs of LIN28 (Supplemental Table S9), suggesting that these interactions have been conserved through evolution. Of the identified LIN-28 targets in *C. elegans*, 46% (537 out of 1168) have human orthologs. Of these, 97 (including *LIN28B*) emerged as targets of LIN28B in a previous study that characterized LIN28 interactions with human transcriptome by PAR-CLIP (Supplemental Table S9; Graf et al. 2013). Overall, in our data set we did not notice a clear enrichment in GO functional categories such as splicing factors or transmembrane protein products as reported by previous studies in mammalian cells (Cho et al. 2012; Wilbert et al. 2012).

### LIN-28 binds a novel site in *C. elegans* pri-*let-7*

As *let-7* miRNA precursors are the most extensively characterized molecular targets of LIN28A and B in vertebrates, we analyzed the interactions of *C. elegans* LIN-28 with genomic regions surrounding miRNAs (Supplemental Table S7). Pri-*let-7* emerges from this analysis as the most significant candidate target, with the lowest adjusted *p*-value (3.87 × 10^−13^) gained from a negative binomial test (see Materials and Methods). Additionally, two other pri-miRNAs appear to be bound by LIN-28 with high probability (Supplemental Fig. S2). One of them, pre-miR-48, is a member of the *let-7* family (Supplemental Fig. S2B). miR-48 and miR-241, another member of the *let-7* family, are encoded <1700 bp apart on the minus strand of chromosome V. Furthermore, LIN-28 binds pre-miR-229, a member of a group of four miRNAs clustered within <1000 bp (miR-64, miR-65, miR-66, and miR-229) on chromosome III (Supplemental Fig. S2A). The proximity of these miRNAs suggests that they might be transcribed as part of single primary transcripts encompassing the entire cluster; in such a scenario, LIN-28 could be involved in modulation of subsequent miR-229 or miR-48 maturation steps, decoupled from miR-64, 65, 66, or miR-241, respectively. Nonetheless, LIN-28 is unlikely to be the sole regulator of miR-48 expression, as a previous study has not detected any difference in levels of mature miR-48 in *lin-28* mutant (Lehrbach et al. 2009). Finally, our CLIP study failed to document an interaction between LIN-28 and miR-85 (data not shown), which is elevated in *lin-28* mutants, suggesting that the previously observed regulatory effect of LIN-28 is likely to take place through an indirect mechanism rather than by direct intermolecular contact (Lehrbach et al. 2009).

In mammals, LIN28 inhibits the expression of *let-7* post-transcriptionally, either by regulating the Drosha-mediated cleavage of pri-*let-7* in the nucleus, or the Dicer-mediated maturation of pre-*let-7* in the cytoplasm (Heo et al. 2008; Newman et al. 2008; Rybak et al. 2008; Viswanathan et al. 2008). In both cases, LIN28 exerts its inhibitory function by binding the terminal loop of pri- or pre-*let-7* in the nucleus or cytoplasm, respectively (Newman et al. 2008; Piskounova et al. 2008; Heo et al. 2009). Within the terminal loop, the Zinc-finger domains specifically recognize a GGAG motif, while the CSD domain interacts with RNA with lower sequence specificity (Nam et al. 2011; Desjardins et al. 2012; Loughlin et al. 2012; Mayr et al. 2012).

**FIGURE 4.**
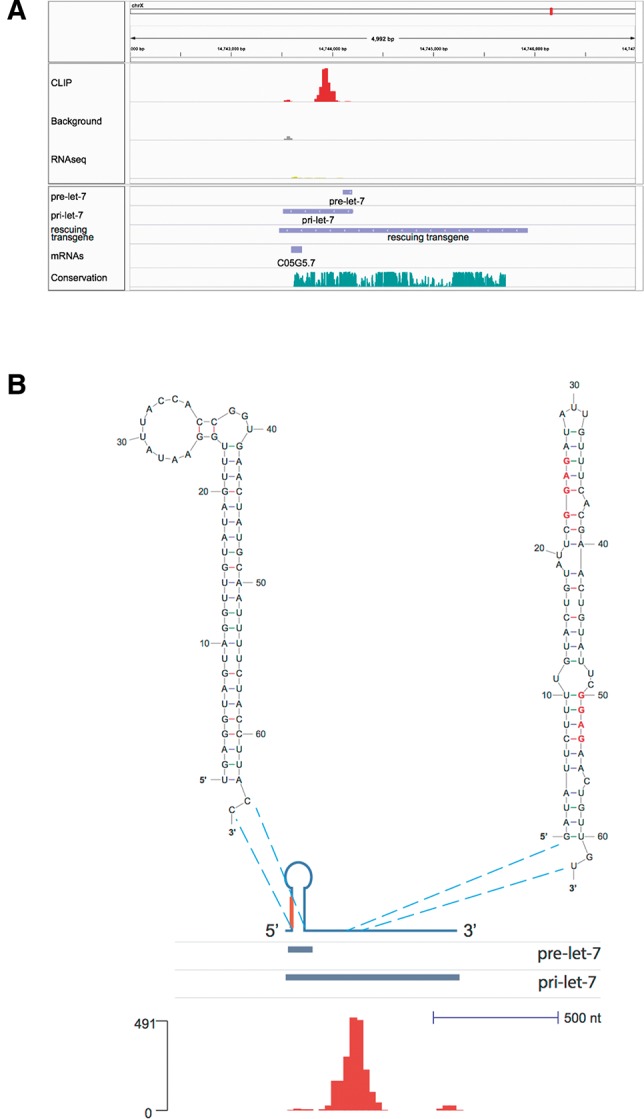
LIN-28 interactions with pri-*let-7*. (*A*) Map of LIN-28 interactions with *let-7* precursors visualized by IGV. The number of reads in each line was normalized by the total number of mapped reads. Pre-*let-7*, pri-*let-7*, and a transgene capable of rescuing the *let-7 mn112* and *mg279* mutations are shown in the *lower* tracks (Reinhart et al. 2000). Since pri-*let-7* is transcribed from the minus strand, its 5′ end corresponds to the *right*-hand end of the bar, while its 3′ end to the *left*. (*B*) The secondary structures of pre-*let-7* and LIN-28 binding site (LBS) predicted using the mfold algorithm, superimposed to a schematic representation of pri-*let-7*, the pre-*let-7*, and pri-*let-7* tracks, and a bar graph representation of the number of reads obtained by LIN-28 HITS-CLIP. For ease of representation, shown is a schematic drawing of pri-*let-7* with annotation tracks and bar graph flipped horizontally compared with *A*, so that the 5′ end is on the *left* side, while the 3′ end is on the *right* side.

In contrast, the terminal loop of *C. elegans* pre-*let-7* lacks a GGAG motif presenting a mystery as to how LIN-28 might bind to *let-7* in this case. The results from our HITS-CLIP experiment do not show an interaction with the terminal loop of *let-7* (Fig. 4A,B). Instead, LIN-28 appears to interact with a region of pri-*let-7* located 170 nt downstream from the predicted 3′ end of pre-*let-7* (Fig. 4A,B). This novel LIN-28 binding site (LBS) contains two GGAG motifs within a region that can be folded to form a weak hairpin structure (predicted folding free energy: −11.70 kcal/mol) (Fig. 4B; Zuker 2003). Two additional GGAG motifs are found within 30 nt of both ends of the LBS.

**FIGURE 5.**
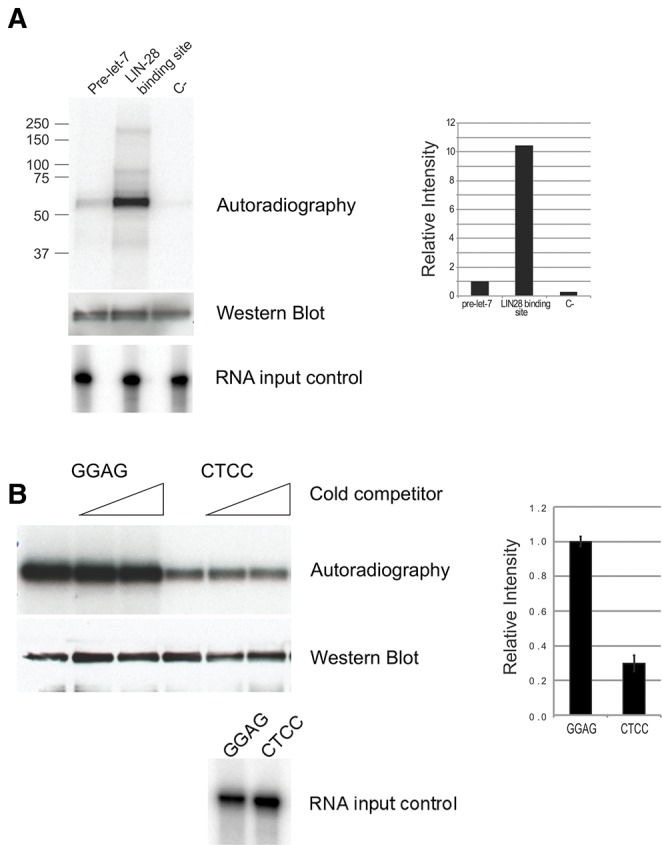
Binding of LIN-28 to pri-let-7 assessed through an in vitro UV-crosslinking assay with radiolabeled RNA. (*A*) Autoradiography showing LIN-28 (fused to GFP, HA, and flag, migrating in SDS-PAGE at ∼55 kDa), expressed in *C. elegans* larvae, immunoprecipitated and UV crosslinked to the indicated P32 body-labeled RNAs (see Materials and Methods for sequences and details). The same filter used for radiography was probed with antibody against HA to verify the presence of equal amounts of LIN-28 (“Western blot”). Labeled RNA corresponding to pre-*let-7*, LBS, and negative control were analyzed by TBE–Urea gel electrophoresis to verify the presence of equal amount of probe and its integrity (“RNA input control”). The panel on the *right* shows a quantitation of the autoradiography by Phosphoimager. (*B*) Interaction of LIN-28 with the LBS or a mutated version of it in which GGAG motifs are changed to CCTC. An in vitro UV-crosslinking assay as in *A* is shown, in which the probe was LBS containing either wild-type GGAG motifs (*right* three lanes) or mutated CTCC (*left* three lanes). The experiment was executed in triplicate for each probe. In the second and third lane of each probes, cold competitor corresponding to negative control (as in *A*) was also included in a 40- and 200-folds molar excess compared with the labeled probe. The same filter used for radiography was probed with antibody against HA (“Western blot”). Labeled RNA corresponding to GGAG or CTCC probes were analyzed by TBE–Urea gel electrophoresis (“RNA input control”). The panel on the *right* shows a quantitation of the autoradiography by Phosphoimager.

We assayed the binding of LIN-28 to the LBS using an in vitro UV-crosslinking assay with radiolabeled RNA (see Materials and Methods for details) that allowed us to use native LIN-28 protein. LIN28 was immunopurified from transgenic LIN-28-HA lysate and incubated with in vitro transcribed, body labeled RNA, and crosslinked with UV light; the covalent protein–RNA complex was then resolved by electrophoresis on a polyacrylamide gel in denaturing conditions. This assay revealed a markedly stronger interaction between LIN-28 and LBS RNA than a RNA of the same length corresponding to the pre-let-7 stem–loop structure (Fig. 5A). A mutation of the GGAG motifs to CTCC within LBS drastically decreased the binding (Fig. 5B). The addition of an unlabeled competitor RNA (with same base composition but scrambled sequence as the “GGAG” probe) to the binding reaction does not affect the binding to LIN-28 of the GGAG nor CTCC mutant probes, demonstrating that both interactions are sequence-specific (Fig. 5B). The incomplete reduction of binding caused by mutation of GGAG repeats, as well as the ability of a CTCC cold competitor to affect binding, albeit with lower efficiency than the GGAG competitor (Supplemental Fig. S3), are consistent with the mode of action of mammalian LIN28, where binding of GGAG with high specificity through ZnF is paired with interaction with RNA through CSD with lower sequence-specificity (Nam et al. 2011; Mayr et al. 2012).

**FIGURE 6.**
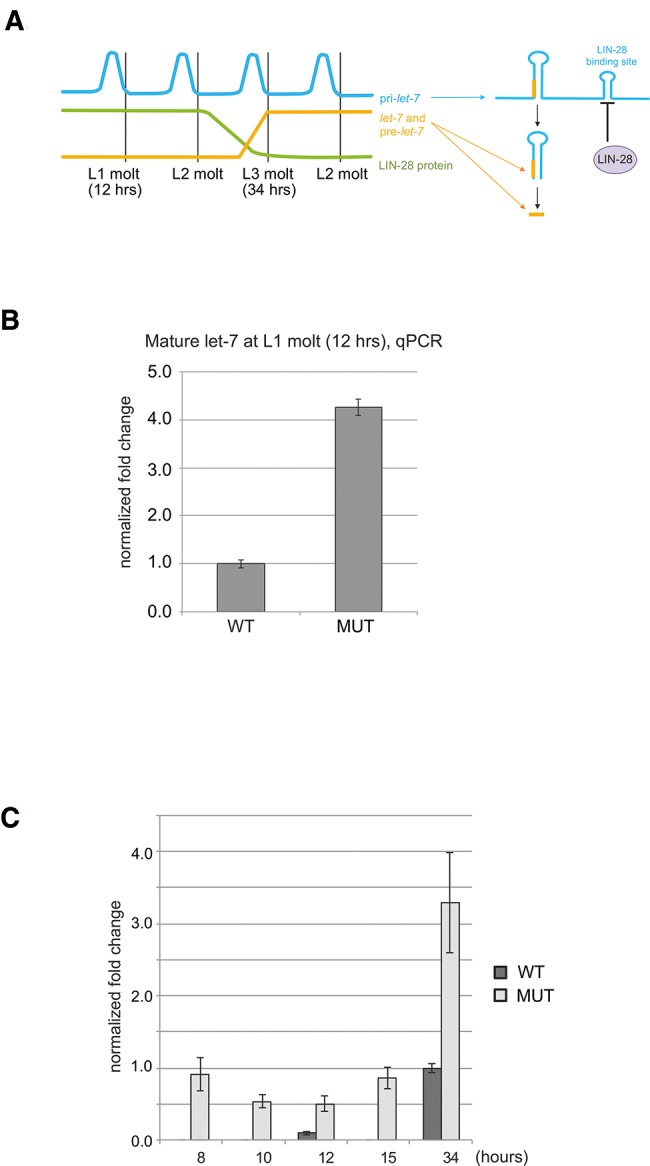
The LBS is required for normal regulation of maturation of *let-7* by LIN-28. (*A*) Schematic representation of the normal pattern of expression of pri-*let-7* (blue), LIN-28 (green), pre-*let-7*, and mature *let-7* (orange) during larval development. (*B*) Mature *let-7* levels detected by RT-qPCR at the time of L1 larval molt in transgenic worms carrying a wild-type *let-7* transgene (WT) or one in which the LBS is deleted (MUT). (*C*) Mature *let-7* levels detected by RT-qPCR at the indicated time points (*x*-axis) in transgenic animals carrying wild-type or mutated transgenes as in *B*.

We assayed the functional importance of the LBS in *let-7* maturation. The expression of *let-7* is characterized by uncoupling of transcription of pri-*let-7* and its post-transcriptional maturation in the larval stages prior to L3: pri-*let-7* is detected at the L1 and L2 molts in the absence of pre-*let-7* and mature *let-7* (Fig. 6A; Van Wynsberghe et al. 2011). In *lin-28* mutants, mature *let-7* is detected from the time of the L1 molt, in agreement with a role of LIN-28 in the uncoupling of transcription and maturation of *let-7* in the early larva (Van Wynsberghe et al. 2011). We reasoned that, since LBS is the site of interaction between LIN-28 and pri-*let-7*, its deletion should result in precocious appearance of mature *let-7*. To test this hypothesis, we generated *C. elegans* transgenic lines carrying low-copy insertion of either a construct containing all the information for proper *let-7* expression (2.5-kb *let-7* rescuing fragment, Reinhart et al. 2000), or a version of the same construct in which the LBS was deleted (Supplemental Fig. S4). Consistent with a role of LBS in mediating repression of maturation, its deletion resulted in a fourfold increase of the levels of mature *let-7* at the time of L1 molt (Fig. 6B). Furthermore, assaying for mature *let-7* by qPCR at 2-h intervals around the time of L1 molt shows that animals carrying the transgene lacking LBS produce an amount of mature *let-7* similar to the amount detected in wild-type transgenes at the normal time of mature *let-7* appearance (34 h, or L3 molt), while mature *let-7* is virtually undetectable in wild type transgenes around the time of L1 molt (8, 10, 12, 15 h) (Fig. 6C). We also noted a threefold increase in the amount of mature *let-7* at the L3 molt time point in the mutated transgene compared with the wild type, despite the same number of copies of transgene integrated in the genome as detected by qPCR (Supplemental Fig. S4). We speculate that other indirect effects downstream from the precociously expressed *let-7* might explain these increased levels, including the demonstrated ability of *let-7* to boost its own expression by recruiting ALG-1 to pri-*let-7* though *let-7* complementary sites (LCS) situated >200 nt downstream from the LBS (Zisoulis et al. 2012). Additionally, upon elimination of LIN-28 by RNAi, we observed a more marked derepression of *let-7* maturation in animals carrying the WT *let-7* transgene than in those expressing the pri-*let-7* form mutated in the LBS (7.45-fold versus 2.74-fold, *P* = 3.75 × 10^−4^, Student's *t*-test) (Supplemental Fig. S5). This finding is consistent with a LIN-28-mediated mechanism of repression of *let-7* maturation through an interaction with the RNA motif identified by HITS-CLIP. Since both wild-type and mutant transgenic lines still carry the endogenous copy of the *let-7* gene, part or the entirety of the 2.74-fold increase in mature *let-7* abundance that we observe in the LBS mutant transgenic line might be explained by loss of LIN28 regulation of the natural gene (Supplemental Fig. S5). Together, these data identify a novel LIN-28 binding site in pri-*let-7* in nematodes.

### The LBS is conserved in other species

**FIGURE 7.**
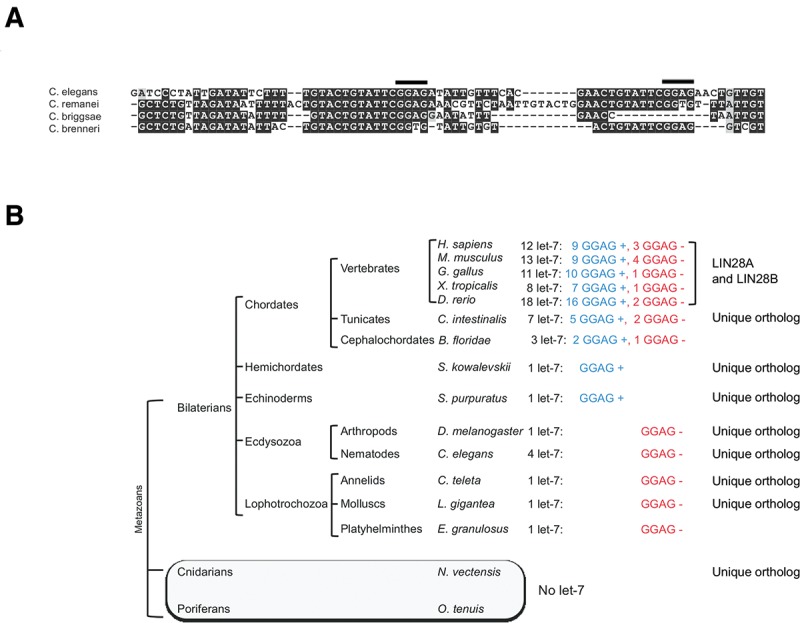
Conservation of the GGAG motifs within the LBS and pre-*let-7* across species. (*A*) Alignment of the LBS region of four nematode species (*C. elegans, C. remanei, C. briggsae, C. brenneri*). (*B*) Phylogenetic distribution of the *let-7* miRNAs in metazoans. For each indicated species, the number of *let-7* genes is indicated in the *left* column (black font). The number of *let-7* genes that have GGAG motifs in their precursor's terminal loop is indicated in the *middle* column (blue font, “GGAG +”), while the number of *let-7* genes that lack such feature is indicated in the *right* column (red font, “GGAG –”). The presence of one or two LIN28 orthologs (*A*,*B*) is indicated in the *rightmost* column.

We analyzed the architecture of pri-*let-7* in species other than *C. elegans*. We noticed a similar arrangement of the sequence elements in pri-*let-7* within nematodes: *C. elegans*, *C. briggsae*, *C. remanei*, and *C. brenneri* lack GGAG motifs within the terminal loop and have elevated sequence conservation within the LBS, including at least one GGAG quadruplet in each species (Fig. 7A). Similarly to *C. elegans*, the candidate LBS sequences in other nematode species are predicted to fold into weak secondary structures (Supplemental Fig. S6A). We further inspected the sequence of the terminal loop of pre-*let-7* across phylogeny for the presence of the tetranucleotide motif GGAG. Among the species we analyzed, Platyhelminthes, Mollusks, Annelids, and Arthropods (*D. melanogaster*), do not have the GGAG motif in the terminal loop of their unique *let-7* gene. The GGAG motif in the terminal loop is present in Echinoderms, Hemichordates, and Chordates. However, in all analyzed Chordate species, where several *let-7* genes are present, at least one of the *let-7* genes does not display the GGAG motif in their terminal loop (Fig. 7B). While the mechanism of regulation by LIN-28 through binding sites in the terminal loop seems prevalent within chordates, the absence of such architecture in some members of the *let-7* family suggest that either some *let-7* isoforms are resistant to LIN28-mediated regulation or that LIN28 binds elsewhere within the primary transcript, in a way similar to our findings in nematodes. In support of the latter model, we detected a predicted stem–loop structure containing three GGAG motifs 172 nt downstream from the precursor stem–loop of human pri-let-7a-3, which does not contain GGAG repeats, in an arrangement reminiscent of *C. elegans* pri-let-7 (Supplemental Fig. S6B).

## DISCUSSION

The RNA-binding protein LIN-28 has long been known to be an important factor in the *C. elegans* heterochronic pathway (Ambros 1989; Slack and Ruvkun 1997), but the nature and scope of its post-transcriptional regulatory program was unknown in the context of a developing organism. In the present study, we utilized in vivo HITS-CLIP to unveil the complete repertoire of in vivo LIN-28-RNA regulatory interactions. The main advantages of this approach derives from the establishment of covalent protein–RNA bonds in vivo, therefore respecting the native intracellular compartmentalization and stoichiometric ratios, followed by highly stringent biochemical purification of the stabilized complexes (Darnell 2012). Our study markedly expands the repertory of known RNA sequences targeted by LIN-28 for regulation. Within the pool of targeted RNAs, we identified in an unbiased way the quadruplet GGAG as the sole enriched sequence motif. This finding is in agreement with the mechanism of RNA recognition by mammalian LIN28, as revealed by the cocrystal structure of murine Lin28 bound to the terminal loop of murine *let-7* (Nam et al. 2011). The latter study, and a number of other biochemical studies of the binding of Lin28 to pre-*let-7*, have shown that the two ZnF domains are engaged in sequence-specific contacts with the GGAG quadruplet, while the CSD interacts with RNA with limited sequence-specificity (Lightfoot et al. 2011; Nam et al. 2011; Desjardins et al. 2012; Loughlin et al. 2012; Mayr et al. 2012). The enrichment of GGAG in our CLIP-generated data set indicates that the sequence-specificity documented in the context of the pre-*let-7* terminal loop is a general feature of LIN-28-RNA interaction throughout the transcriptome and across species. Similar conclusions have been drawn from HITS-CLIP studies in vertebrate cells (Cho et al. 2012; Wilbert et al. 2012). However, the low information content of such a short motif suggests that other factors, such as RNA structure and cooperative or competitive binding with other proteins also play a role in defining the sites of in vivo interactions between LIN-28 and RNA.

Consistent with the role of LIN-28 as a regulator of animal development, the pool of novel identified targets includes a large number of genes functionally classified as developmental genes. While the present study reveals a large number of novel molecular targets whose functional interactions with LIN-28 will be the subject of future investigation, the validity of the data set is supported by the presence of a number of heterochronic genes that have long established genetic relationships with *lin-28*. We detected an interaction between LIN-28 and *lin-14* mRNA, a regulator of the early phases of larval development whose expression is modulated by *lin-28* (Arasu et al. 1991). Furthermore, we detected binding to *lin-46* mRNA, a gene epistatic to *lin-28* (Pepper et al. 2004). Finally, HITS-CLIP confirms the direct interaction of LIN-28 with *let-7* that has been extensively documented in vertebrate cells (Heo et al. 2008; Newman et al. 2008; Viswanathan et al. 2008).

In striking contrast with all previous evidence, we did not detect binding with the terminal loop of pri- or pre-*let-7*, but with a site located within pri-*let-7* about 170 nt downstream from pre-*let-7*. Unlike the vertebrate *let-7* precursors that have been analyzed thus far, the terminal loop of *C. elegans let-7* lacks GGAG quadruplets, while this motif is present in multiple copies in the downstream site we identified by CLIP. A similar architecture of *let-7* primary transcripts, characterized by absent GGAG in the terminal loop and downstream bona fide LIN-28 binding sites, is shared among the known nematode pri-*let-7* sequences. Furthermore, GGAG quadruplets are absent from the terminal loop of the available pre-*let-7* sequences of Platyhelminthes (*E. granulosus*), Mollusks (*L. gigantea*), Annelids (*C. teleta*) and *D. melanogaster*, which have one *let-7* gene each. In Chordates, which generally have several *let-7* genes, GGAG quadruplets are absent from the terminal loops of at least one of their *let-7* genes. These observations suggest that an ancestral mode of regulation of *let-7* maturation by LIN-28 through a binding site located outside the terminal loop, present in nematodes, might have been maintained throughout evolution, alongside the more recently evolved regulation through direct inhibition of Dicer or Drosha through binding of the *let-7* terminal loop.

## MATERIALS AND METHODS

### HITS-CLIP

HITS-CLIP experiments were performed as follows. *C. elegans* transgenic strains carrying a single copy of a modified *lin-28* gene, encoding a fusion GFP, flag, HAHA at the carboxy-terminus, were generated by bombardment. The expression of the transgene at the proper time and place was verified by RT-PCR, Western blot and by its ability to fully rescue the phenotype of the *lin-28(n719)* mutant strain. Liquid cultures of staged, fed L1 larvae (containing about five million animals) were harvested by centrifugation, washed in M9 solution, and treated with UV in a Stratalinker (3.6 mJ/cm^2^). Subsequently, worms were lysed with zirconia beads by three 20-sec cycles in a MP Fastprep 24 in buffer A (20 mM HEPES pH 7.4, 150 mM NaCl, 0.1% SDS, 0.5% deoxycholate, 0.5% NP40, 20 mM EDTA and 20 mM EGTA). The lysate was cleared by ultracentrifugation (100,000*g*, 30 min). Subsequent steps were performed as described previously, with few modifications (Ule et al. 2005; Jensen and Darnell 2008). LIN-28/RNA complexes were purified with a commercial antibody anti-HA (HA-7, Sigma H3663) conjugated with Dynabeads (Life Technologies 112-01D). During the subsequent washing steps, the complexes were treated with an optimized amount of micrococcal nuclease to achieve an average RNA size of ∼70 nt, as estimated by gel electrophoresis. A 5′ end adapter (5′-/5AmMC6/AGGGAGGACGAUGCGG-3′) was ligated overnight. Following SDS-PAGE purification and proteinase K treatment, a 3′ end adapter (5′-P-GUGUCAGUCACUUCCAGCGG-Pmn) was ligated, and Reverse Transcription/PCR was performed (forward primer: 5′-AATGATACGGCGACCACCGACTATGGATACTTAGTCAGGGAGGACGATGCGG-3′, reverse primer: 5′-CAAGCAGAAGACGGCATACGACCGCTGGAAGTGACTGACAC-3′). Libraries thus prepared were sequenced in an Illumina HighSeq 2000 machine using primer 5′-CTATGGATACTTAGTCAGGGAGGACGATGCGG-3′. RNA-seq libraries were performed from total RNA purified from L1 larvae reared the same way, following oligo(dT) selection, according to the standard Illumina protocol.

### RNA-CoIP, RT-qPCR

RNA co-IP, followed by qPCR where performed as follows: *C. elegans* larvae were harvested, UV-treated and lysed as described above. Following clearing by ultracentrifugation and preincubation with beads conjugated with mouse IgG, protein–RNA complexes were purified using anti-HA antibodies (HA-7, Sigma H3663) conjugated with Dynabeads (Life Technologies 112-01D). After overnight incubation at 4°C, complexes were washed three times with buffer A (see above), three times with buffer B (20 mM HEPES pH 7.4, 300 mM NaCl, 0.1% SDS, 0.5% deoxycholate, 0.5% NP40, 20 mM EDTA and 20 mM EGTA) and once with buffer E (100 mM Tris–HCl, pH 7.4, 50 mM NaCl, 10 mM EDTA). During these washes, the complexes were treated with DNAse (Turbo DNAse, Ambion). Finally, RNA was eluted by treatment with proteinase K followed by two phenol–chloroform extractions and precipitation. Reverse transcription was performed using random hexamers and Superscript III (Life Technologies). Mature *let-7* was detected using a Taqman Assay (Life technologies). Quantitative PCR was conducted in a Roche Lightcycler LC480. See Supplemental Table S8 for primer sequences.

### Protein–RNA in vitro cross-linking

RNA was transcribed in vitro using T7 RNA polymerase and a 134-bp DNA template corresponding to the LIN-28 binding site identified by CLIP (WT), a version of the same sequence where the four GGAG sequences were mutated to CTCC (MUT), a scrambled sequence with the same nucleotide composition as WT (C-), and the pre-*let-7* distal loop (pre-*let-7*) (see Supplemental Table S8 for sequences). The transcription mix contained cold GTP and P32-labeled GTP (in a 2.8:1 molar ratio). In vitro transcribed RNA was gel-purified before the assay. *C. elegans* larvae protein extract was prepared as described above, using a different lysis buffer (20 mM HEPES, pH 7.4, 150 mM NaCl, 0.2% NP40, 3 mM MgCl_2_, 1 mM DTT). Equal counts of RNA (roughly corresponding to 20 fmol) were heated at 65°C for 5 min, then incubated with *C. elegans* larvae protein extract (300 µg of total protein) for 10 min at 30°C in 100 µL, in the presence or absence of cold competitor RNA. At the end of the incubation, the reaction mix was crosslinked for 15 min on ice in a 48-wells plate in a Stratalinker. After immune-purification, the protein–RNA complexes were washed and treated with micrococcal nuclease (NEB, diluted 1:100) for 10 min at 37°C. After further washes, the protein complexes were eluted in SDS-PAGE sample buffer at 80°C for 10 min, resolved on a 4%–12% Bis–Tris gel (Biorad) and transferred to a nitrocellulose membrane. The membrane was exposed to a phosphoimager and to film.

### Data processing

Reads from both CLIP and RNA-seq experiments were mapped to the *C. elegans* genome version WS190/ce6 using Novoalign (http://www.novocraft.com) with parameters “-F ILMFQ -t 85 -l 25 -s 1 -o SAM -r None”. The program can remove adapters at the read ends and allow identification of substitutions and small indels in the reads. To exclude ambiguous regions, we only considered reads that mapped to exon regions and miRNA regions. Since most of the genes in Refseq database in UCSC genome browser (http://genome.ucsc.edu/) lack UTR annotation, we extended 200 bp at 5′ end and 750 bp at 3′ end based on the known average UTR length (95% quantile of UTR length, 5′ UTR: ∼200 bp, 3′ UTR: ∼450 bp) in Wormbase (http://www.wormbase.org/) and the mapped tag density around coding regions. Then the overlapping exon regions were concatenated to generate the target exon regions for subsequent analysis. For miRNAs, pre-miRNA coordinate information was downloaded from MirBase (version 13.0), and then extended 1000 bp up and downstream to generate putative pri-miRNAs. To avoid confusion coming from reads of exon regions, the extended regions overlapped with exons defined above were cut to the position right after the exons, and the miRNAs were discarded if pre-miRNA regions overlapped with exons. Reads that mapped to the exons or miRNAs were extracted and summarized for 150-bp windows. Since our CLIP-seq data were generated from strand-specific sequencing, it was summarized for each of the forward and reverse strands separately. On the other hand, RNA-seq data was generated from two-stranded sequencing, so the two strands were combined to give the final counts for each window.

### Binding site identification in mRNA regions

#### CIMS analysis

To accurately obtain potential binding sites with crosslinking induced mutations, we first examined the mutation patterns induced by cross-linking in CLIP-seq. In order to determine the subtype of the mutations representing cross-linking sites, we summarized and analyzed mutations as three independent types—substitution, deletion, and insertion. Mutations were clustered if they were mapped at the same position. For mutations longer than 1 bp, only the first base was considered. To distinguish CIMS from sequencing errors, we ranked the mutation positions with a Binomial test (Equation 1) from the hypothesis testing whether the proportion of reads with mutation in the position is significantly higher than that in the whole genome. The *p*-values were adjusted for multiple testing using Benjamini–Hochberg (BH) method (Benjamini and Hochberg 1995).

(1) pvalue(a|y,p)=∑x≥a(yx)px(1−p)y−xwherep=no. of mutations typeno. of reads×read length,

where *a* is the number of mutations at the position and *y* is the total number of reads mapped to that position. We also filtered ambiguous mutations using the following criteria. First, sequencing technology usually introduces errors on repeated tandem sequences (e.g., region containing a sequence of same nucleotides, such as TTTT), so we extracted the surrounding regions of mutation positions and excluded those on nucleotide tandem sequences with at least 5 repeats. Second, to avoid PCR amplification biases, we required mutation clusters containing at least three uniquely mapped mutations (e.g., from three unique reads).

After filtering, the top 500 mutation positions ranked with BH adjusted *p*-values (≤0.05 required) in each mutation type were extended 15 bp upstream and downstream, and then the sequences were extracted from UCSC genome browser and subjected to the MEME algorithm to identify motifs (Bailey et al. 2009) with parameters -mod zoops -nmotifs 3 -minw 4 -maxw 8 -dna -maxsize 500000. To see the enrichment levels of motifs, we searched the motif identified from deletion clusters in all mutation positions using the FIMO algorithm (Grant et al. 2011) with parameters --output-pthresh 5e-3 --motif 1 –norc --max-stored-scores 500000. The resolution of CIMS analysis on binding site identification was obtained by considering motif distance from positions of deletion clusters.

#### Peak analysis

A combined parametric model with dynamic Poisson and negative binomial regression was used to obtain the putative binding sites from tag counts. RNA-seq data was used as a matching control for CLIPseq.

In the dynamic Poisson model, background Poisson mean in each window for CLIPseq is locally estimated using the read counts in nearby windows of the RNA-seq sample according to gene and exon annotations. Since we concatenated overlapping exons to generate windows, a window may belong to more than one gene or exon region. So we chose to use the maximum parameter from genes, exons and surrounding regions as the parameter for each window in the dynamic Poisson model, as shown in Equation 2. In the model, RNA-seq tag counts were first normalized based on the total read count ratio in exon regions of CLIP-seq and RNA-seq.

(2) $$p(x_{i}|\lambda_{\text{i}})=\frac{\lambda_{i}^{x_{i}}}{x_{i}!}e^{-\lambda_{i}},\lambda_{i}=\max[\underset{\;j=1,\ldots ,J}{\max}(\lambda_{gj}),\;\underset{k=1,\ldots ,K}{\max}(\lambda_{ek}),\;\underset{l=1,\ldots ,L}{\max}(\lambda_{sl})],$$

where λ_*i*_ is the Poisson parameter estimated from RNA-seq data and used to calculate *p*-value for the *i*th window; λ_*gj*_ is the parameter for gene *j* that the *i*th window belongs to; λ_*ek*_ is the parameter for exon *k* that the *i*th window belongs to; and λ_*sl*_ is the parameter from surrounding region *l* of the *i*th window. The surrounding region is defined as the windows on the exon island that the *i*th window belongs to, and the exon island is defined as the nonoverlapping and concatenated exon regions on the genome. We chose λ_*i*_ to be the maximum value among all these parameters to control false-positive peak identifications. *x*_*i*_ and λ_*i*_ were normalized by the window length.

We also used a negative binomial regression method (Equation 3) to capture the relatedness between CLIPseq and RNA-seq globally and the dispersion of CLIPseq given the RNA-seq. Log-transformed RNA-seq counts were used in the model and maximum likelihood estimation was used to gain the parameters.

(3) $$\begin{array}{l} \log\;E(X_{i}|R_{i}=r_{i})=\log\mu (r_{i})=a+b\;\log(r_{i})\\ p(x_{i}|r_{i},a,b,\alpha )=\frac{\Gamma (x_{i}+\alpha^{-1})}{x_{i}!\Gamma (\alpha^{-1})}{\left(\frac{\mu (r_{i})}{\mu (r_{i})+\alpha^{-1}}\right)}^{x_{i}}{\left(\frac{\alpha^{-1}}{\mu (r_{i})+\alpha^{-1}}\right)}^{1/\alpha }, \end{array}$$

where *x*_*i*_ is the count of CLIPseq in window *i*, *r*_*i*_ is the count of RNA-seq in window *i*, *a*, and *b* are regression coefficients, and α is dispersion parameter.

After we estimated parameters for each model, the *p*-value was calculated as *P*(*X* > *x*_*i*_ | θ), where *x*_*i*_ is the read count in the *i*th window and θ are the parameter estimates. Finally, the *p*-values from the two models were combined by Fisher's method as Equation 4. Then the windows were ranked by the combined *p*-values. To further control the false positives, we only considered the windows with the number of unique positions covered by reads more than the third quartile of that among all windows.

(4) $$p\;\text{value}(x_{i})=P(X>x_{i}|\lambda_{i})\times P(X>x_{i}|r_{i},a,b,\alpha ).$$

Top 500 peak windows were extended 100 bp upstream and downstream and then subjected to MEME to search for motifs with parameters -mod zoops -nmotifs 3 -minw 4 -maxw 8 -dna -maxsize 500000. The motif with the best *E*-value was selected as the motif identified by peak analysis. Top 2000 peak windows were selected for binding features analysis, such as binding distribution on transcripts, resolution of binding sites and GO analysis. The resolution of binding site identification by peak analysis was obtained by considering the distance of window center to high confident mutations (top mutations from deletions and substitutions) defined binding sites. We considered real peaks those that emerged in each independent experiment. Despite the difference in sequencing depth, the identified binding sites and read distribution pattern are very similar in two repeats, as shown in Figure 1B. Almost all the peaks found in the less deep data set (referred to as CLIP1) are also present in the deeper data set (CLIP2) as well. The main difference is that the CLIP2 covers wider genomic regions, but most of those regions are covered by fewer than 10 tags, which suggests they may represent background.

### Binding site identification in microRNA regions

Since RNA-seq is specifically designed to study mRNAs, it is not suitable to be used as the matching control for microRNA regions. Thus, one-sample analysis without control was applied on microRNA regions. To consider the possible overdispersion of the CLIP-seq data, we used a negative binomial model (Equation 5) to identify the binding sites in microRNA regions. The parameters were estimated using maximum likelihood estimation method. *p*-values were adjusted with Benjamini–Hochberg (BH) method.

(5) $$p(x|\mu ,\alpha )=\frac{\Gamma (x+\alpha^{-1})}{x!\Gamma (\alpha^{-1})}{(}^{\frac{\mu }{\mu +\alpha^{-1}})x}{\left(\frac{\alpha^{-1}}{\mu +\alpha^{-1}}\right)}^{1/\alpha }.$$

### GO analysis

The Refseq IDs of the genes corresponding to the top 1500 binding sites (Supplemental Table S2) were analyzed with the Functional Annotation Clustering Tool of the David website (http://david.abcc.ncifcrf.gov) with the following parameters: Classification Stringency: Highest; Similarity Term Overlap: 3; Similarity Threshold: 1; Initial and Final Group Membership: 3; Multiple Linkage Threshold: 0.50; Enrichment Threshold EASE: 1.0; Display: Benjamini.

## SUPPLEMENTAL MATERIAL

Supplemental material is available for this article.

## Supplementary Material

Supplemental Material
