# Supplementary material for: A novel mechanism of LIN-28 regulation of let-7 microRNA expression revealed by in vivo HITS-CLIP in C. elegans
Source: RNA. 2015 May;21(5):985–96. doi: 10.1261/rna.045542.114 (PMC4408804; doi:10.1261/rna.045542.114)
Supplement: Supplemental Material [file supp_21_5_985__index.html]

A novel mechanism of LIN-28 regulation of let-7 microRNA expression revealed by in vivo HITS-CLIP in C. elegans — A novel mechanism of LIN-28 regulation of let-7 microRNA expression revealed by in vivo HITS-CLIP in C. elegans — Supplemental Material 

# A novel mechanism of LIN-28 regulation of *let-7* microRNA expression revealed by in vivo HITS-CLIP in *C. elegans*

## Supplemental Material

**Files in this Data Supplement:**

- Supp Figure S1.pdf
- Supp Figure S2.pdf
- Supp Figure S3.pdf
- Supp Figure S4.pdf
- Supp Figure S5.pdf
- Supp Figure S6.pdf
- Supp Fig Legends.docx
- Supp Table S7.xls
- Supp Table S3.xls
- Supp Table S2.xls
- Supp Table S8.xls
- Supp Table S9.xls
- Supp Table S6.xls
- Supp Table S4.xls
- Supp Table S1.xls
- Supp Table S5.xls
