# Supplementary figures and images for: A novel mechanism of LIN-28 regulation of let-7 microRNA expression revealed by in vivo HITS-CLIP in C. elegans
Source: RNA. 2015 May;21(5):985–96. doi: 10.1261/rna.045542.114 (PMC4408804; doi:10.1261/rna.045542.114)

# A

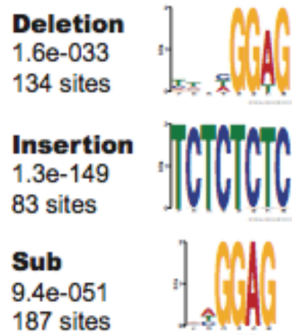

# B

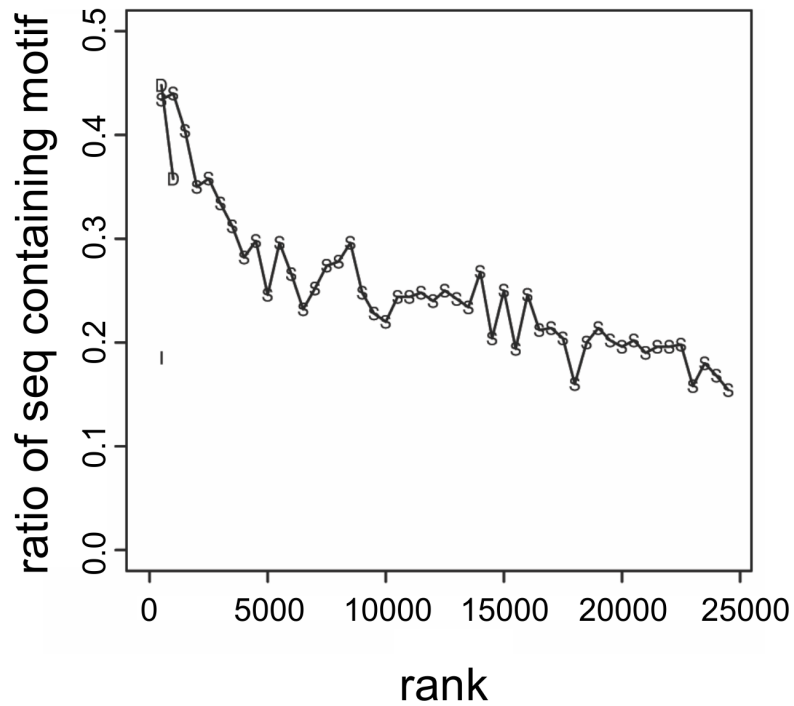

# C

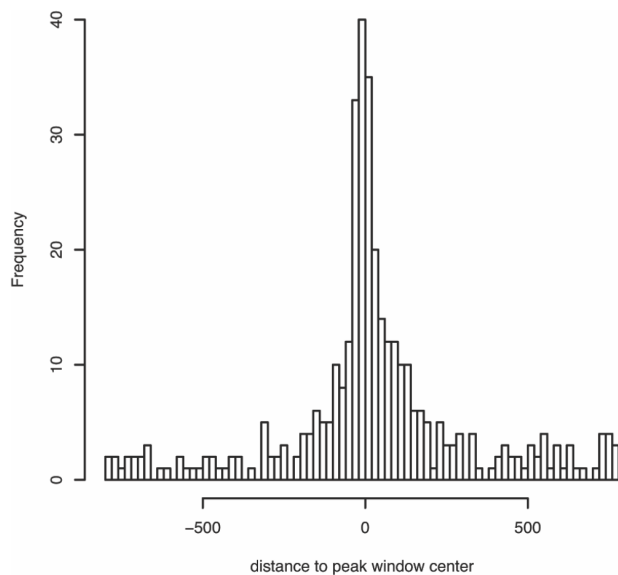

# D

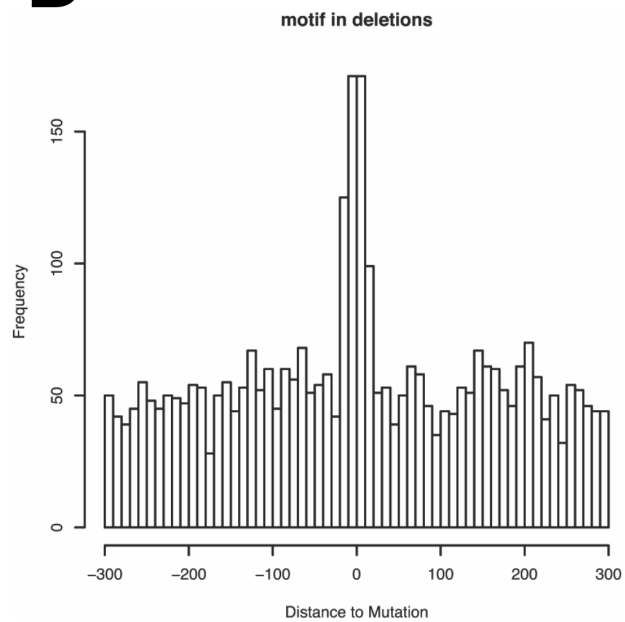

Supplement: Supplemental Material [file supp_045542.114_Fig_S1.pdf]

A

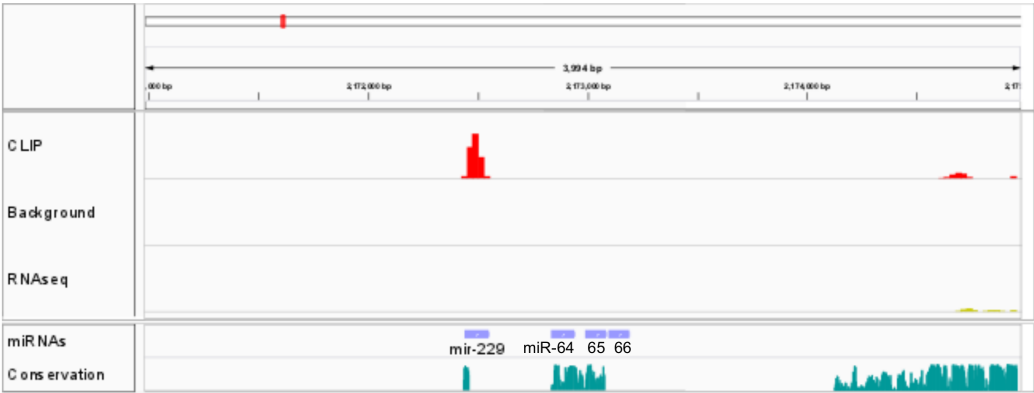

B

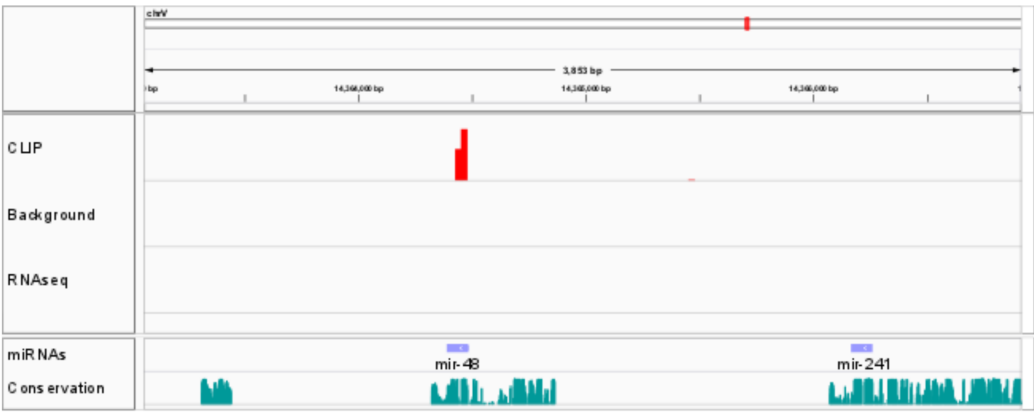

Supplement: Supplemental Material [file supp_045542.114_Fig_S2.pdf]

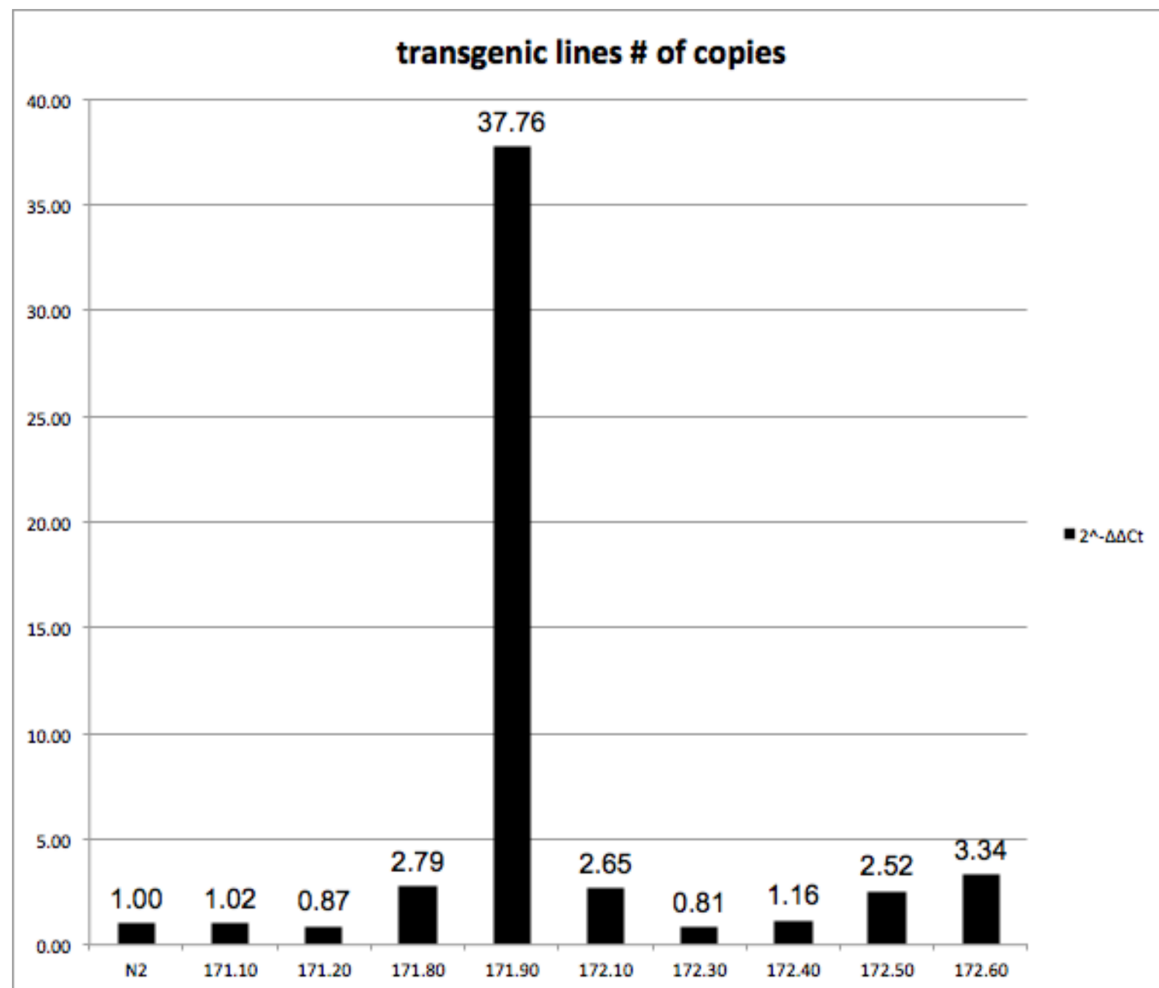

Supplement: Supplemental Material [file supp_045542.114_Fig_S4.pdf]

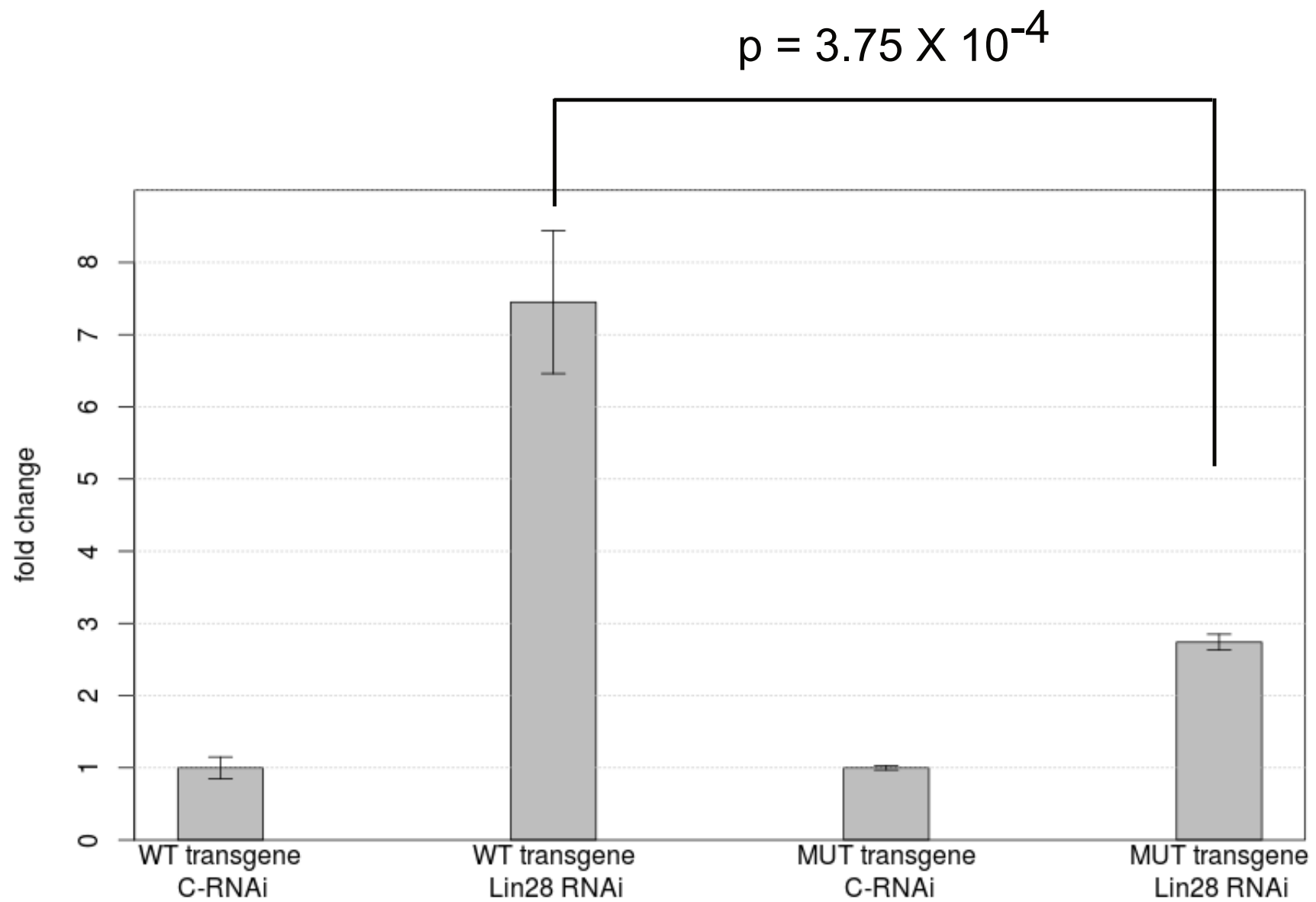

Supplement: Supplemental Material [file supp_045542.114_Fig_S5_cmyk.pdf]

# A

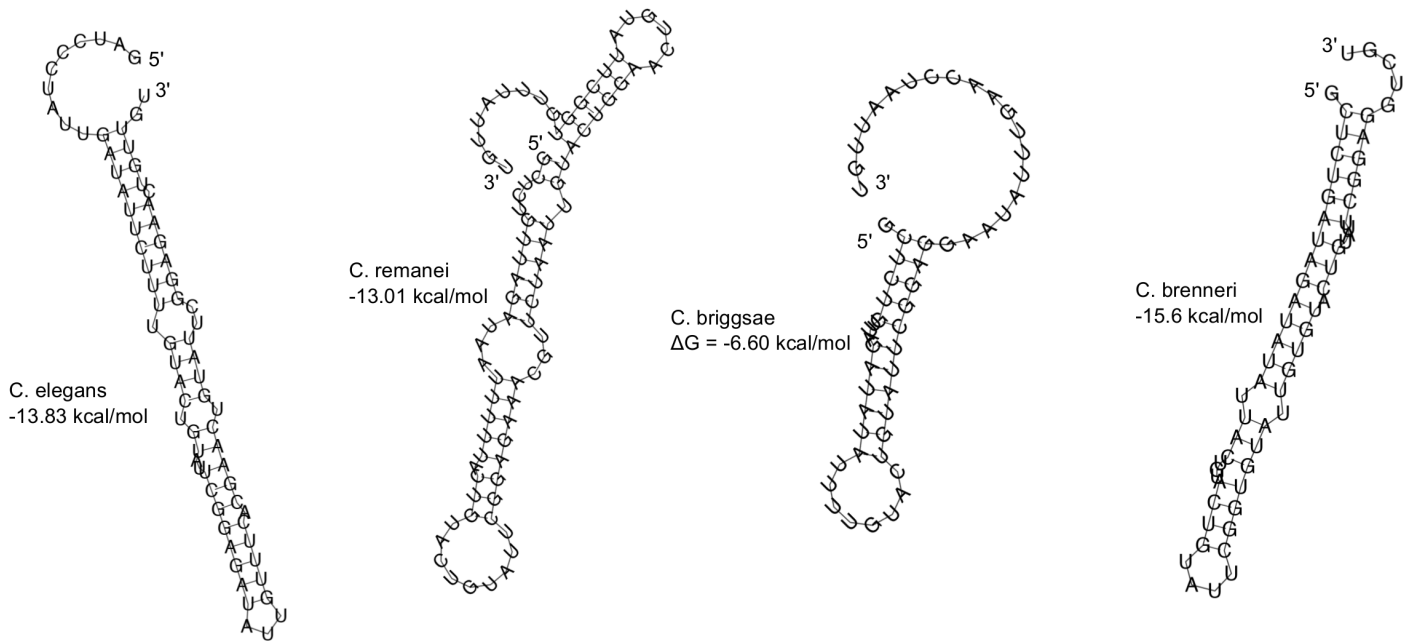

# B

*H.sapiens* pri-let-7a-3

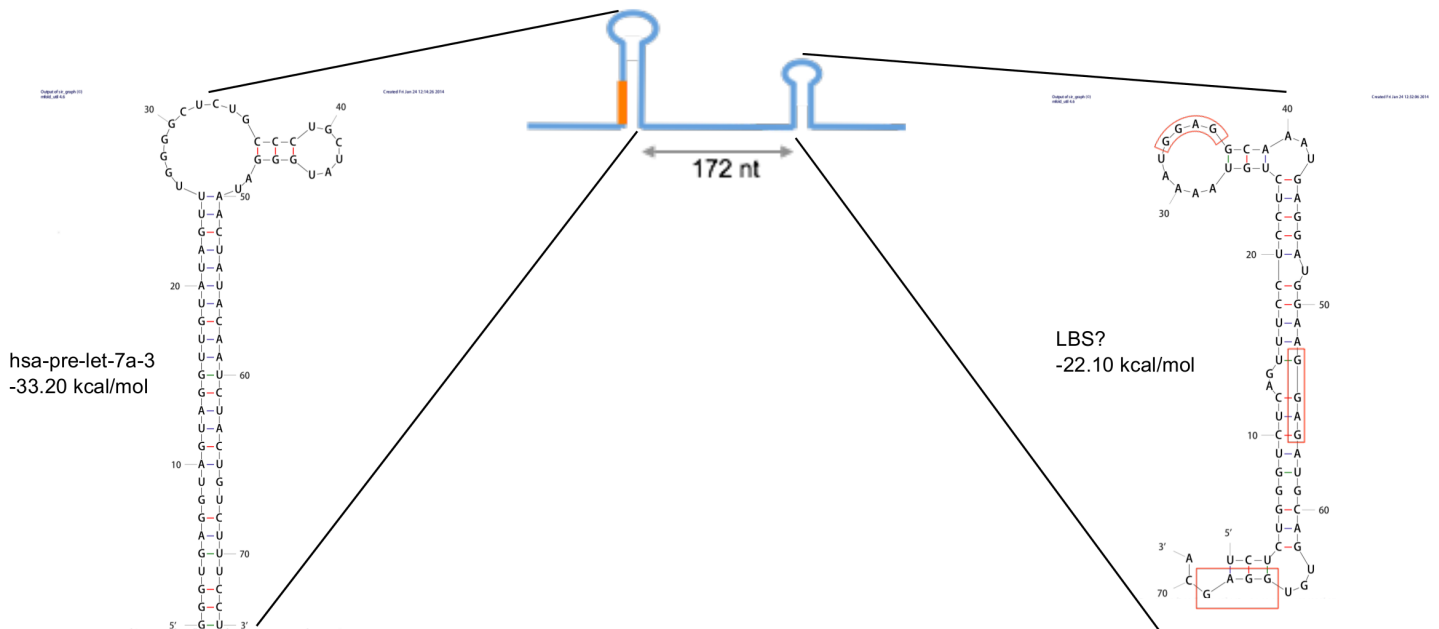

Supplement: Supplemental Material [file supp_045542.114_Fig_S6.pdf]
