# Supplementary material for: A novel mechanism of LIN-28 regulation of let-7 microRNA expression revealed by in vivo HITS-CLIP in C. elegans
Source: RNA. 2015 May;21(5):985–96. doi: 10.1261/rna.045542.114 (PMC4408804; doi:10.1261/rna.045542.114)
Supplement: Supplemental Material [file supp_045542.114_SuppFigLegends.docx]

Supplementary Figure legends:

Fig. S1: CIMS analysis. A) Motifs discovered by MEME analysis within the binding sites dataset defined by deletions, Insertions and substitutions within the CLIP2 dataset alone. B) Deletions (D), substitutions (S) and Insertion (I) were ranked by a binomial test (see Methods), and the presence of the GGAG motif within a stretch of 30 residues surrounding the point mutation was assessed by MEME analysis. The ratio of such sequences containing a GGAG motif is plotted as a function of the mutation ranking. C) Distribution of the distance of GGAG motif from the peak window center. D) Distribution of the distance of the GGAG motif from the CIMS (deletion).

Fig. S2: LIN-28 interactions with miR-229 and miR-48. A) Map of LIN-28 interactions with the miR-229, miR-64, 65, 66 cluster visualized by IGV. Number of reads in each line was normalized by total number of mapped reads. B) Map of LIN-28 interactions with the miR-48, miR-241 cluster visualized by IGV.

Fig. S3: Binding of LIN-28 to LBS is inhibited in the presence of cold competitor RNA. A) Autoradiography showing LIN-28 immuno-purified and cross-linked to P32 body-labeled wild type extended LBS RNA as in figure 5B. Labeled wild type LBS RNA was cross-linked to LIN-28 in the presence of increasing amounts of cold WT (GGAG) or mutant (CTCC) RNA competitor (molar ratio cold to labeled RNA: 0, 40, 200). The same filter used for radiography was probed with antibody against HA to verify the presence of equal amounts of LIN-28 (‘Western Blot’). B) Cold competitor RNA (‘scrambled’ used in experiment shown in figure 5B) were analyzed on a TBE-Urea polyacrylamide gel to verify the presence of equal amount of probe and its integrity.

Fig. S4: Quantification of the transgene copy number in the transgenic lines used to assay the effects of LBS deletion on let-7 maturation. Transgenic lines were generated by bombardment in *unc-119* background. Four stable lines were obtained with the unaltered construct (171.1, 171.2, 171.8, 171.9) and five with the LBS deletion (172.1, 172.3, 172.4, 172.5, 172.6). The copy number of transgene was quantified by qPCR on genomic DNA, using wild type animals for normalization (N2). Lines 171.8, 172.1 and 172.5 were used for experiments.

Fig. S5: Abundance of mature let-7 detected by Taqman qPCR in transgenic animals carrying a WT pri-let-7 transgene (WT) or one in which the LBS was deleted (MUT). In both these transgenic lines, the endogenous *let-7* gene was still present. RNA was extracted 12 hours after hatching and the abundance of mature let-7 and pri-let-7 were assessed by Taqman qPCR and SYBR green qPCR, respectively. Bars represent fold change of *let-7* abundance in *Lin-28* RNAi relative to negative control RNAi, normalized for pri-let-7 abundance.

Fig. S6: Conservation of the LBS among different species: A) Hypothetic secondary structure of LBS region in *C. elegans*, *C. remanei*. *C. briggsae* and *C. brenneri*. B) *Homo sapiens* pri-*let-7a3* does not have GGAG motifs in the terminal loop of the precursor (left side of the figure), and, similarly to *C. elegans*, has a folded structure about 170 nucleotides downstream, with three GGAG motifs.
