# Supplementary material for: A novel mechanism of LIN-28 regulation of let-7 microRNA expression revealed by in vivo HITS-CLIP in C. elegans
Source: RNA. 2015 May;21(5):985–96. doi: 10.1261/rna.045542.114 (PMC4408804; doi:10.1261/rna.045542.114)
Supplement: Supplemental Material [file supp_045542.114_Fig_S3.pdf]

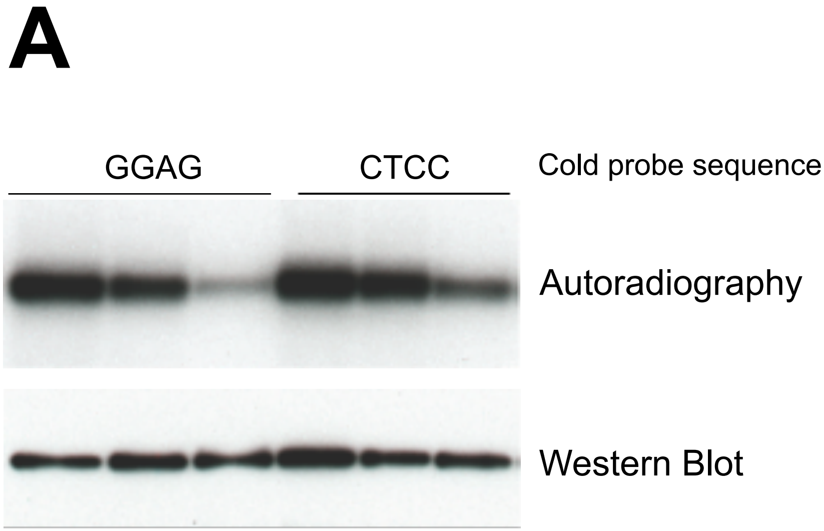
A
GGAG
CTCC
Cold probe sequence
Autoradiography
Western Blot

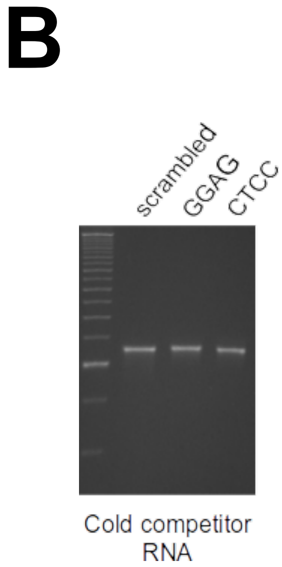
B
scrambled
GGAG
CTCC
Cold competitor RNA
